# Modelling Psychological Responses to the Great East Japan Earthquake and Nuclear Incident

**DOI:** 10.1371/journal.pone.0037690

**Published:** 2012-05-30

**Authors:** Robin Goodwin, Masahito Takahashi, Shaojing Sun, Stanley O. Gaines

**Affiliations:** 1 Department of Psychology, School of Social Sciences, Brunel University, Uxbridge, Middlesex, United Kingdom; 2 Faculty of Humanities, Yamaguchi University, Yamaguchi, Japan; 3 School of Journalism, Fudan University, Shanghai, China; University of Sydney, Australia

## Abstract

The Great East Japan (Tōhoku/Kanto) earthquake of March 2011was followed by a major tsunami and nuclear incident. Several previous studies have suggested a number of psychological responses to such disasters. However, few previous studies have modelled individual differences in the risk perceptions of major events, or the implications of these perceptions for relevant behaviours. We conducted a survey specifically examining responses to the Great Japan earthquake and nuclear incident, with data collected 11–13 weeks following these events. 844 young respondents completed a questionnaire in three regions of Japan; Miyagi (close to the earthquake and leaking nuclear plants), Tokyo/Chiba (approximately 220 km from the nuclear plants), and Western Japan (Yamaguchi and Nagasaki, some 1000 km from the plants). Results indicated significant regional differences in risk perception, with greater concern over earthquake risks in Tokyo than in Miyagi or Western Japan. Structural equation analyses showed that shared normative concerns about earthquake and nuclear risks, conservation values, lack of trust in governmental advice about the nuclear hazard, and poor personal control over the nuclear incident were positively correlated with perceived earthquake and nuclear risks. These risk perceptions further predicted specific outcomes (e.g. modifying homes, avoiding going outside, contemplating leaving Japan). The strength and significance of these pathways varied by region. Mental health and practical implications of these findings are discussed in the light of the continuing uncertainties in Japan following the March 2011 events.

## Introduction

The Great East Japan (Tōhoku/Kanto) earthquake of March 11^th^ 2011, measuring 9.0+ (Richter scale), was the largest earthquake in that country’s geologically turbulent history, and one of the largest earthquakes in recorded history [1]. The earthquake was accompanied by a large tsunami, the two events killing more than 19,000 people [2]. This tsunami caused severe damage to the nuclear reactors at Fukushima, seriously affecting all six reactors with meltdowns occuring in three. This nuclear incident was declared “Level 7” by the Japanese Ministry of Economy, Trade and Industry, the highest level on the International Nuclear and Radiological Event Scale [3]. This places the Fukushima incident as being of comparable severity to the 1986 Chernobyl meltdown. In this paper we examine how Japanese people across Japan reacted to the earthquake and nuclear incident, considering variations in risk perception across individuals and locations, and their behavioural consequences.

Japan has been subject to a large number of natural disasters, including earthquakes, tsunamis, typoons and volcanic eruptions [3]–[5]. Research in Japan has indentified a wide range of stress-related responses to these disasters, including anxiety, depression, and sleeping disorders [4]. Work on earthquakes in Kobe (1995) and Niigata (2006) has examined post-traumatic stress and its manifestations in other health outcomes (e.g. suicide rates) [6]–[9]. This knowledge helped prepare mental health support services within Japan following the March 11^th^ events [10], with relevant professional groups, such as the Japanese Society for Psychiatry and Neurology, introducing immediate countermeasures within days of the earthquake [5]. However, it has been increasingly recognised – in Japan and elsewhere – that psychological responses to such major societal events vary considerably across individuals and groups [5], [11], [12]. As yet, however, few attempts have been made to examine individual and situational variations in response to such large-scale disasters [13].

Our research aimed to directly model individual, personal differences in risk perceptions, and the implications of this for subsequent behaviour. Drawing on transaction appraisal [14], [15], psychodynamic [16] and risk theories [17] we suggest that individual characteristics (such as values) and social networks will differentially influence assessment of the Great Japan earthquake and nuclear risks. Individual values can act as significant predictors of risk perception. Previous work has found values that emphasise tradition, conformity and security (collectively termed ‘conservation values’) to be positively correlated with worries over a range of life domains [18]. In a study of the threat posed by the H1N1 [swine flu) pandemic, those who scored highly on conservation values were also most likely to be concerned about being infected by the pandemic [19]. In our current study we hypothesise that scores on conservation will be positively correlated with perceived risks from both the earthquake and the nuclear incident. However, perceptions of threat do not exist in a ‘vacuum’, with those around us likely to influence our risk perceptions [20]. This may be particularly significant at a time when wider communication may be limited, as was the case immediately after the Great Japan earthquake [21]. Those whose ‘normative networks’ (friends, family) express anxiety about these incidents are more likely to be stressed, through a ‘social contagion’ effect [17]. Further, individuals vary in the degree to which they believe that they can control risks [22], with those individuals who believe they are less able to control their safety likely to perceive themselves at higher risk [23], [24]. Recently, Japanese commentators have partialled those responding to the Fukushima incident into ‘safety’ versus ‘risk’ ‘junkies’, contrasting those who underestimate risks with those who overestimate the risks that followed the nuclear incident [25]. Risk perceptions about the Fukushima nuclear plants are also likely to be influenced by trust in governmental advice about the nuclear meltdowns, with this trust correlating negatively with perceived radiation risks [26].

In this paper we predict that earthquake and nuclear risk perceptions will correlate with two sets of outcomes. Earthquake risk perception will positively correlate with those activities most likely to ameliorate the consequences of an earthquake, namely keeping an earthquake kit and making modifications to the home. Similarly, nuclear risk should positively correlate with particularly precautionary activities; namely avoidance of particular foods, avoiding going outside, and wearing a face mask to avoid radiation. Stocking up on food, and contemplating leaving Japan are relevant to both risk events, and will be positively correlated with both risk assessments (earthquake and nuclear risks). In line with previous research into risks [27] we also hypothesise that social dislocation is likely to have an impact on risk perception and behavioural change. The 2011 earthquake had its centre in rural areas, and such dislocation may be particularly great in such locations, where there is strong attachment to locations and housing [28]. We compared data across the three regions of Japan in our data set (Western Japan, Tokyo and Chiba, Miyagi), anticipating the greatest risk perception to be in the area most affected by the earthquake (Miyagi).

### Overview of this Paper

In this paper we collected data from young people two to three months after the Great East Japan earthquake. Data was collected on individual values, the anxieties of friends and family, perceived control over risk, trust in government advice, perceived risk, and actions taken as a result of the earthquake and nuclear incidents. We collected this data three different regions of Japan: Miyagi, close to the epicentre of the earthquake [2] and the area where there was the largest loss of life and property damage (approximately 100 km, or 62 miles, from Fukushima); Tokyo and the neighbouring Chiba prefecture (approximately 220 km from the Fukushima plants) and ‘Western Japan’ (Yamaguchi and Nagasaki, approximately 1000 km from the plants). Such an analysis allows us to explore risk perceptions and behaviours across those regions differentially impacted by the March 11^th^ earthquake and nuclear incident.

## Methods

### Participants and Procedure

Respondents were 844 university students attending seven Universities located in the three areas listed above (Miyagi, *N* = 235; Tokyo and Chiba, *N* = 247; Western Japan (Yamaguchi and Nagasaki), *N* = 362). All data was collected over 12 days between May 30^th^ and June 11^th^ 2011 (i.e. 11–13 weeks after the earthquake). Data collection dates did not differ significantly across sites: site collection dates were 30^th^ May –7^th^ June (Western Japan); 7^th^ June –9^th^ June (Tokyo) and 9^th^ June –11^th^ June (Miyagi).

This study received ethical approval from The Department of Psychology, Brunel University Ethics Board as well as agreement from the Faculty of Humanities at Yamaguchi University. In line with the procedures of the Japanese Social Psychological Society all participants provided verbal informed consent. Written consent was not obtained, as verbal consent is more consistent with the procedures used and approved in Japan. All participants completed an anonymous paper and pencil questionnaire in Japanese during class time, given to them by their class tutors. Participants were, of course, free to not answer questions or remove themselves from the study at any time without penalisation.

### Measures

Details of the questionnaire items are provided in Table 1. Questions included demographic items (age, sex, region), and an indication of previous personal loss. The questionnaires also included four sets of predictors of perceived risk. The first set consisted of three items assessing conservation values (*M* = 5.24, *SD* = 1.25, Cronbach α = .70) [29]. To assess normative influence we included two items, one measuring normative concerns about an earthquake (*M* = 2.70, *SD* = .84), the second normative concerns about the nuclear incident (*M* = 2.76, *SD* = .85). Our third predictor (perceived control over safety) similarly included two items (control over safety following an earthquake; *M* = 2.00, *SD* = .55, control over a nuclear incident, *M* = 1.40, *SD* = .56). while our fourth predictor assessed trust in government’s advice about the radiation risks (*M* = 2.05, *SD* = .71). Perceived risk itself was measured by two items, one assessing perceived risk from a further earthquake, a second risk from a future nuclear incident. Behavioural responses to the earthquake or nuclear incident were assessed by three sets of items, the first set specific to earthquake risk (two questions), the second specific to nuclear risk (three questions), and the third applicable to both risks (two questions).

**Table 1:** Questionnaire items.

Measure	*N* item	Items	Scale range	Scale points
Demographics	2	Age, sex	Actual age	
Previous personal losses	1	Has someone close to you been seriously injured/killed in an earthquake?	Yes/no	2
Risk predictors and perceived risk				
Conservation Values	3	How important are the values of: security, conformity, tradition	“opposed to my values” to “of supreme importance”,	9
Normative concern	2	How concerned about the 11^th^ March earthquake(Fukushima nuclear incident) are your friends and family?	not at all concerned, a little concerned, quite concerned,very concerned	4
Control over safety	2	How much control over safety do you have during anearthquake (nuclear incident)?	not at all controllable, a litle controllable, very controllable	3
Trust in government’s advice	1	How much do you trust the government’s adviceabout radiation risks?	don’t trust at all, trust only a little, trust quite a lot, completely trust	4
Perceived risk	2	How much risk do you think there is of a future earthquake(nuclear incident) seriously affecting your safety?	no risk at all risk, not much risk,some risk, a great of risk	4
Response to earthquakes or the nuclear incident				
Earthquake responses	2	Since the 11th March earthquake have you kept an emergencykit? Since the 11th March earthquake have you modifiedyour house to help avoid injury during earthquakes?	Yes/no	2
Nuclear risk responses	3	As a result of the radiation risk did you a) avoid certainfoods and drinks as a result of the radiation risk? b) avoid goingoutside or try to limit the time you were outdoors to avoidradiation? c) wear masks to avoid radiation?	not at all, once or twice,occasionally, very often	4
Both earthquake and nuclear responses	2	As a result of the earthquake/nuclear incident did you a) stockup on food b) think about leaving Japan, at least for a while	a) yes/no. b) not at all; I considered this; yes, seriously.	2, 3

### Statistical Procedure

Following descriptive analysis of respondents across sites, we examine significant differences by region in both risk perception and behavioural responses using analysis of variance, controlling for sex and age. We then perform a structural equation analysis using AMOS (version 18.0) to examine predictors and outcomes of both earthquake and nuclear risk perception. First, data distribution were examined and multivariate normality evidenced in light of Mardia’s coefficient [30]. Because of the small amount of missing data, a random missing pattern was assumed and missing data entries imputed with means. We then analyzed covariance matrices with the maximum likelihood estimation method. To achieve an explanatory and parsimonious model, we compared nested models with reference to a Chi-square test. We fitted a fully mediated model with 7 single outcome variables (e.g. housing modification, stocking food, etc. as single dependent variables) (see Figure S1). This conceptual model showed a poor fit to the pooled data (*x^2^* = 1441.598, df = 66, p<.001, GFI = .757, CFI = .286, RMSEA = .157, SRMR = .145) [31]. Moreover, the tested conceptual model did not converge for two of the three subsamples. Further exploratory analysis revealed that the residuals of outcome variables are highly correlated with each other. Instead of correlating residuals arbitrarily, we turned to item parceling to account for the commonalities between residuals and to simplify the model [32]. To inspect the interrelational structure of the behavioral outcomes, we conducted hierarchical cluster analysis with Ward’s clustering method [33]. As compared to traditional principal components analysis and factor analysis, this method is suitable for classifying items into groups when the number of items is not large. After examining the dendrogram and the agglomeration schedule, we identified a three-cluster solution. The first cluster, labeled as nuclear acts, consisted of three items, “avoid certain foods and drinks,” “avoid going outside,” and “wear masks” (Cronbach α = .73). The second cluster, labeled as ‘earthquake acts’, consisted of two items, “kept an emergency kit’ and “modifying homes” (*r* = .31). The last cluster consisted of a single item, “considered leaving Japan.” We utilized cluster scores by averaging items within each cluster to model the relationships among variables. After fitting the model to the pooled data first, we moved to comparing the model fit across three different sites, using a step-by-step multiple-group comparison procedure [30].

## Results

### Regional Differences in Demographics

Respondent characteristics from the three regions were first analysed across age and sex. There were significant effects for age and sex across the samples. There were more male respondents in our data set from Miyagi (*x* ^2^ (2) = 23.97, *p*<. 01; 59% of respondents were male in Miyagi compared to 41% in Western Japan and 39% in Tokyo/Chiba). Our Tokyo/Chiba respondents were also significantly older (*F* (2, 838) = 69.78, *p*<.001: *M* age = 23 for Tokyo/Chiba, *M* age = 19 in each of the other two data sites).

### Individual Risk Perceptions and Behavioural Outcomes

Given a four point scale (*no risk at all risk, not much risk, some risk, a great of risk*) participants in general perceived ‘some’ risk of a future earthquake or nuclear incident seriously impacting on their safety (59% and 48% respectively gave this response; *M*s 2.84 (SD.66) and 2.75 (.73) on a 4-point scale, high scores indicating higher risk: see Table 1 for scale points). 29% of respondents indicated keeping an emergency kit since the earthquake, 13% modifying their house, 31% stocking up on food/drink. Almost half our respondents (43%) reported avoidance of some foods or drink, although only 20% reported any avoidance of going outside because of radiation, and only 22% wore masks at any time to minimise radiation risks. Eleven percent had contemplated leaving Japan following the nuclear incident.

### Regional Analysis: Risk Perception and Behavioural Responses

**Figure 1:**
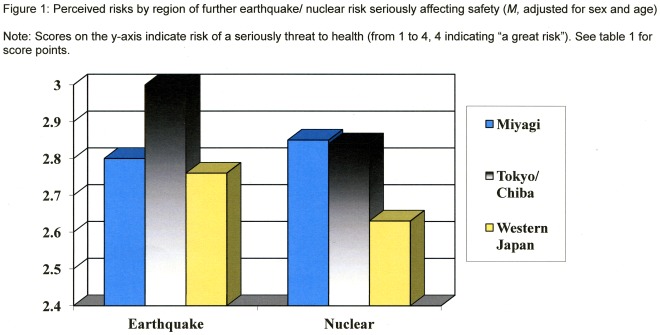
Perceived risks by region of further earthquake/nuclear risk. Note: Scores on the y-axis indicate risk of a seriously threat to safety (from 1 to 4, 4 indicating “a great risk”). See table 1 for score points.

Analysis by region indicated significant differences in both risk perception and behavioural responses (see Figure 1). Controlling for age and sex, respondents in Tokyo/Chiba perceived a greater risk of an earthquake seriously affecting their safety (*F* (2, 831) = 9.59, *p*<.001), although it was those in Miyagi that were most likely to report having someone close to them killed or seriously injured in an earthquake (17% reported this in Miyagi, compared to 5% in Tokyo/Chiba and 2% in Western Japan, *x^2^* (2) = 53.72, *p*<.001). Respondents in Western Japan were significantly less likely to perceive a risk from a nuclear incident than those in the other two areas (*F* (2, 833) = 8.66, *p*<.001). Respondents in Western Japan were less likely to report avoiding foods or drink (*F* (2, 833) = 29.39, *p*<.001) or going outside (*F* (2, 833) = 28.74, *p*<.001), and were less likely to consider leaving Japan (*F* (2, 833) = 6. 98, *p*<.001). Those in Western Japan were also less likely to report keeping an earthquake kit (*x^2^* (2) = 138.58, *p*<.001), making changes to the house (*x^2^* (2) = 120.49, *p*<.001) or stocking up on food since the earthquake and nuclear incident (*x^2^* (2) = 168.52, *p*<.001).

### Structural Analyses

**Figure 2:**
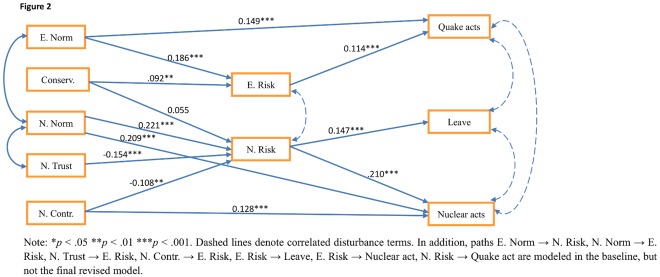
Final revised structural model. Note: *p<.05, **p<.01, ***p<.001. Paths E. Norm → N. Risk, N. Norm → E. Risk, N. Trust → E. Risk, N. Contr. → E. Risk, E. Risk → Leave, E. Risk → Nuclear act, N. Risk → Quake act are modeled in the baseline, but not the final revised model.

Following the preliminary steps described above (‘statistical procedure’), we first tried to fit a fully-mediated baseline model with parceled items, which showed a poor fit (*x^2^* = 415.11, df = 25, p<.001, GFI = .913, CFI = .610, RMSEA = .136, SRMR = .101) (Figure S2). After further model revision, in which we allowed for direct effects between norms and relevant acts and between nuclear control and nuclear acts, the final revised model fitted the pooled sample data satisfactorily (*x^2^* = 80.53, df = 27, p<.001, GFI = .982, CFI = .947, RMSEA = .048, SRMR = .046) (Figure 2). In this model, earthquake norms and conservation significantly correlated with earthquake risk, with those high on conservation more likely to perceive a greater risk from an earthquake, and those whose friends and family perceive a greater threat are also likely to adjudge greater earthquake risk. Similarly, those whose friends or family perceive greater nuclear risk were also likely to perceive such a risk; those who do not trust in government, and those with a lesser sense of control over a nuclear threat, were likely to be more anxious about the nuclear risk. Earthquake risk predicted ‘quake acts’ (keeping an emergency kit and modifying houses); nuclear risk predicted both nuclear acts (avoiding certain food and drinks, avoiding going outside and wearing masks) and considering leaving Japan. There were also direct pathways between earthquake norms and earthquake acts (those whose families and friends were anxious about the earthquake were more likely to perform the ‘quake acts’), as well as between nuclear control and nuclear acts, with those who feel they have the most control more likely to perform these acts.

### Structural Model by Region

**Figure 3:**
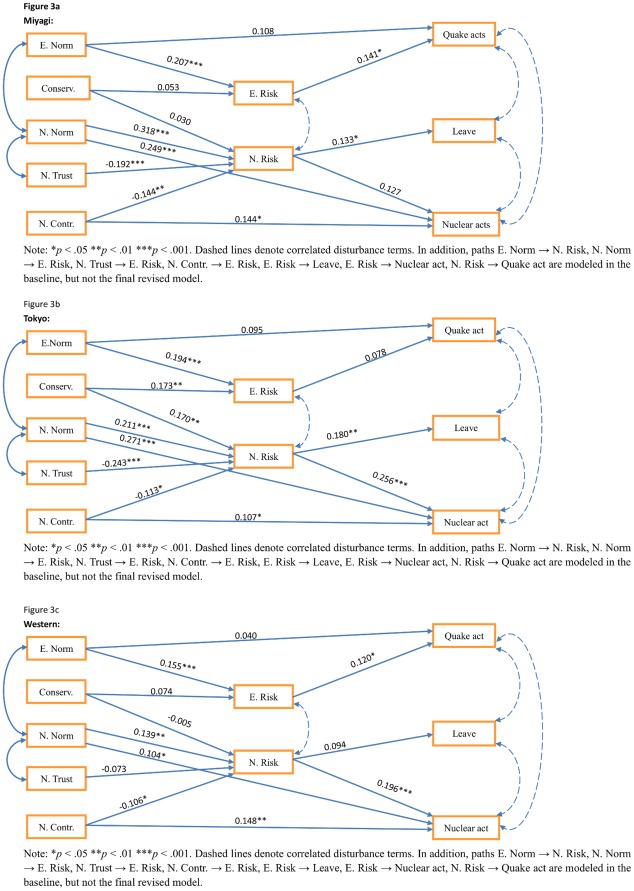
Final structural model, by region. **Figure 3a:** **Final structural model, Miyagi only** Note: *p<.05, **p<.01, ***p<.001. Paths E. Norm → N. Risk, N. Norm → E. Risk, N. Trust → E. Risk, N. Contr. → E. Risk, E. Risk → Leave, E. Risk → Nuclear act, N. Risk → Quake act are modeled in the baseline, but not the final revised model. **Figure 3b:** **Final structural model, Tokyo only** **Figure 3c:** **Final structural model, Western Japan only.**

We fitted the revised model to each subsample, with model fit satisfactory for each sample (see Table 2). Then, allowing all parameters to vary across groups, the final model was fitted to the three groups simultaneously (*x^2^* = 145.34, df = 81, p<.001, GFI = .969, CFI = .930, RMSEA = .031, SRMR = .052). The resultant fit indices suggested that the same structural configurations can be fitted to the three different samples [30], and by constraining all regression path coefficients to be equal across sites, we fitted a constrained model (*x^2^* = 192.21, df = 105, p<.001, GFI = .958, CFI = .905, RMSEA = .031, SRMR = .066). Further comparative tests however indicated that not all the structural coefficients were uniform across the three sites (Δ *x^2^* = 46.87, Δdf = 24, p<.01)(see Figure 3a–c; for standardized residual matrices see Table S1).

**Table 2:** Model Fit Across Sites.

Model	χ^2^	df	p	*X* ^2^/df	SRMR	GFI	CFI	RMSEA
Miyagi	32.66	27	.209	1.21	.052	.974	.975	.030
Tokyo/Chiba	64.01	27	.000	2.37	.072	.954	.890	.072
Western Japan	48.67	27	.006	1.80	.052	.975	.938	.047
Cut-off^31^				3	<.10	>.90	>.90	<.10

As can be seen from the figures, normative indicators of earthquake or nuclear risk were significant positive predictors of earthquake and nuclear risk across all the sites, with regression weights ranging from.16 (Western Japan) to.21 (Miyagi) for the relationship between earthquake norms and earthquake risks, and from.14 (Western Japan) to.32 (Miyagi) for nuclear norms and nuclear risks. Normative nuclear risks also had a direct positive relationship with nuclear related actions across sites (regression weights ranging from.10 (Western Japan) to.27 (Tokyo)). In addition, across all sites, perception of control over a nuclear risk was significantly negatively related to both anxiety about a nuclear risk (weights ranging from −.11 (Western Japan) to −.14 (Migayi)), and positively related to nuclear actions (from.11 (Tokyo) to.15 (Western Japan)). However, trust in governmental advice was significantly (negatively) related to nuclear risk perception in only Miyagi (−.19) and Tokyo (−.24), while conservation values were only significantly (positively) related to either earthquake or nuclear risk perceptions in Tokyo (.17, for both). Earthquake risk was significantly positively related to earthquake acts in Miyagi (regression weight = .14) and Western Japan (.12); nuclear risk was significantly positively related to nuclear acts in Tokyo (.26) and Western Japan (.20). Finally, nuclear risk was positively related to contemplating leaving Japan in Miyagi (.13) and Tokyo (.18), but not Western Japan (.10). We discuss these variations in our conclusions below. Although included in our overall model (Figure 2), earthquake norms were not significant direct predictors of earthquake acts in any of the three individual sites.

## Discussion

The great East Japan earthquake, the subsequent tsunami, and the continuing uncertainty about nuclear leakages provide serious challenges to the citizens of that country. Consistent with our expectations, we found significant relationships between four antecedent variables (respondent’s individual values, normative concerns of their friends and family, their sense of control over the nuclear threat, and their trust in governmental messages) and their perceptions of risk. These risk perceptions in turn predicted changes in preventive actions (keeping an earthquake kit, modifying living quarters) and avoidance behaviours (avoiding certain foods or going outside, wearing masks, contemplating leaving the country). Our results, however, indicated significant differences in regional responses to the threat, with greater anticipated risk of a future earthquake in Tokyo than Miyagi or Western Japan, while behavioural changes were larger in areas most affected by the March 2011 events. Our findings also indicated significant regional variations in the relationship between values, trust in government and risk perceptions, and between risk perceptions and behavioural outcomes. We discuss each of these in more detail below.

### Perceived Risks After the Great East Japan Earthquake

First, let us consider our findings from across the samples. Consistent with previous work on the psychological predictors of anxiety and risk perception [18], [22], we found that those individuals who hold values that stress security, tradition and conformity (‘conservation’ values) feel particularly threatened by changes in their surroundings, and are more likely to be anxious about the threat from an earthquake or nuclear incident.

As anticipated, normative perceptions of threats correlated significantly with perceived risks: during widespread threat, individuals seek to reduce their anxieties by sharing their concerns with others [34], but in the process may ‘catch’ the emotional anxieties of their confidants [35]. Indeed, Japan is a relatively ‘collectively orientated’ culture, where shared representations may be more influential than in more individualistic societies [36]. A sense of control over the nuclear threat allows preparedness [37]; lack of control over this risk was correlated with nuclear risk perception, and had a direct impact on nuclear acts (avoiding certain foods and drinks or going outside, wearing masks) in each of the three locations. However, in our structural model a perception of control over the earthquake threat did not have a significant impact on perceived earthquake risk or the direct actions taken to minimise this risk. This may be because natural disasters such as earthquakes in Japan fit in well with a belief in *Shouganai* (“it cannot be helped”). Controllability therefore may be less of a significant predictor of anxiety or avoidant activities when faced with such naturally occuring threats.

Our findings also suggest that anxiety about future earthquakes and anxiety about nuclear threat may lead to different behavioural outcomes. Although relatively few houses collapsed as result of the 2011 Great East Japan earthquake [21], in our data household modifications and the keeping of an emergency kit were predicted by anxiety about future earthquakes; avoiding foods, going outside and wearing face masks by nuclear risk. Further, it was the nuclear risk, and not a continuing earthquake threat, that led to stocking up of food and drink, reflecting perhaps the continuing uncertainty about food security and safety following the Fukushima incident [38]. It was the nuclear risk too, not continuing earthquake hazards, that predicted a willingness to consider leaving Japan. The manmade threat of nuclear power has been seen as a particular ‘dread’ risk, with potentially severe ‘ripple effects’ that act as harbingers of further catastrophies [39].

### Regional Variations in Modelling Risk Perception and Behavioural Responses

Our analyses suggested significant regional differences in both levels of risk perception and behavioural responses, and the relationship between the variables in our structural model (Figure 2). We consider each in turn.

In our data, it was the residents of Tokyo and nearby Chiba that were most anxious about further earthquakes, not those nearer the epicenter of the March 11^th^ earthquake and the following aftershocks. This may be due to several reasons. Those who live or study in ‘high risk’ areas may find themselves in a ‘dissonant state’, where their desire for safety may clash with their potential ‘high risk’ habitat [40]. Alternatively, the relatively high anxiety about earthquake risk found in Tokyo may result from the much discussed threat of a great Kanto earthquake devastating the crowded city [41]. A lack of experience with nuclear plant failure contrasts with the more familiar threat of earthquakes in Japan, and may explain the equally high levels of threat felt by those living in both Miyagi and Tokyo/Chiba. In addition, those living near the nuclear power plant may fear being seen as ‘tainted’ and contaminated. A fear of those affected by nuclear disasters (*hibakusha*) was reported in earlier generations, amongst those exposed to the A-bomb [4]. Following the 2011 nuclear incident, those lacking noncontamination certificates were initially denied access to shelters [21].

Turning to regional variations in our model (figures 3a–3c), individual values were only significant in predicting risk perceptions in Tokyo, a large city often viewed as more ‘individualistic’ than other parts of Japan. This is consistent with other work suggesting that the influence of values and other individual attributes is weaker on behavioural outcomes in more collectivistic settings [42]. Trust in the government in relation to the nuclear risk was a significant predictor of anxiety about nuclear risk in those locations most affected by the earthquake (Miyagi, and to a lesser extent Tokyo/Chiba). This might reflect the importance of such information for those living closest to the nuclear incident, as well as the significantly smaller variance in governmental trust scores in the least affected region. Perceived earthquake risk had a significant impact on related actions (keeping an emergency kit, modifying houses) in Miyagi and Western Japan : this may result from both the impracticality of household modifications in a large city such as Tokyo, as well as significantly less variance in earthquake risk scores in Tokyo/Chiba. The inability of nuclear risk to predict contemplating leaving Japan in Western Japan is likely to reflect ‘floor effects’ in scoring: nuclear risk concerns were low in Western Japan and showed less significant variation than in the other regions. Finally, the lack of a significant relationship between nuclear risk concern and nuclear actions in Miyagi may be a consequence of the immediate focus on the earthquake rather than the nuclear incident in that region. Notably, this was the region with the highest mortality rate following the earthquake [2], and the one where our participants were most likely to report having someone close to them killed or severely injured by an earthquake.

### Implications and Mental Health Interventions in Japan

What are the mental health implications of the 2011 earthquake? Despite the stress –related responses that often follow earthquakes and other natural disasters, resilience to such disasters has been reported in several studies in Japan [4], [9]. The indications so far are also of considerable resilience and adaptation following the Great East Japan earthquake [43]. This may reflect a familiarity with earthquake threat in the region most affected: the smaller Iwate-Miyagi Inland Earthquake (2008) allowed some to prepare their emergency responses [43]. In addition, cultural values can moderate hazard perceptions, and help frame explanations for particular events [24]. The response to the Great East Japan earthquake may therefore reflect a broader optimistic bias that has been reported amongst the Japanese following negative life events [44].

For all this, particular groups are likely to remain vulnerable, with earthquakes liable to trigger multiple negative life events [6], and with delayed dysfunction often a consequence of natural disasters [45]. To deal with this, a number of studies have suggested post-event interventions that utilise both family and existing community resources to reduce distress [28], [45], [46]. At the same time, while strong levels of social support following natural disasters in Japan have been related to positive health outcomes [47], our findings also suggest that sharing risk respresentations amongst close others can encourage worry and fear. Indeed, the strong relationship between the anxieties of families and friends and personal risk estimates in our findings underlines the risk of “emotional contagion” between groups during a time of collective concern [35]. Those advising on the use of such community resources must therefore be aware of the potential importance of ‘shared knowledge’ at a time of continuing uncertainty. Our value findings also suggest practical implications for motivating particular groups towards appropriate behaviors, although we recognize that these interventions may be most effective in the more individualist settings of large cities. While our data suggests that those who value the more ‘collectively orientated’ conservation values may be sufficiently concerned to take particular preventive actions, those with opposing values (such as those who emphasise their own ‘self-direction’] [18] are less likely to react to risk warnings and to modify their behaviours accordingly. Given that many individuals have the power to change their environment, and in doing so increase their resilience (e.g. by attaching bookcases to a wall) [48], safety campaigns need to focus on motivating relevant interventions by stressing the individual self-fulfillment that can be gained from such activities. Finally, while older populations with enduring chronic diseases may be particularly vulnerable immediately following a natural disaster [5], [49]–[51], other populations may demonstrate increased vulnerability over time, as individuals and families try to rebuild their lives [6]. Younger people, who have usually had less experience of traumatic life events, may find longer-term adjustment difficult [9], [28]. This is particularly likely to be the case if employment opportunities are negatively affected by the earthquake [28]. Particular experiences (e.g. loud noses) can act as stress triggers for all age groups, even if these experiences are apparently unrelated to the earthquake or tsunami [4], [52]. Reporting psychological stresses may however be particularly difficult in smaller rural communities, where there is stigma against confessing psychiatric disorder [4], [5]. Those planning interventions in these areas need to be aware of such barriers; future work could profitably follow our young student sample in the most affected areas as they attempt to cope with their losses and rebuild their lives.

The media are likely to play an important role in risk perception ‘making sense’ of traumatic events, informing the public about necessary reactions, as well as providing information about the continuing threat to the wider public [22], [53]. In Japan, trust in the government’s handling of the nuclear incident fell in the months following March 2011 [54]. Given the relationship between trust in governmental advice and anxiety about the nuclear risk in those areas most affected by the Great East Japan Earthquake, the media can play an important role in responsibly explaining official risk estimates to an increasingly skeptical population [55]. Our results also indicate that giving the population a greater sense of control over the nuclear threat is likely to significantly change both perception of risk and socially (and economically) significant actions, such as avoiding certain foods. It should also be noted, however, that information from different media sources may produce different outcomes. Data on media use following the Fukishima incidents suggest significant differences between those who use anonymous internet bulletin boards (e.g. BBS 2 ch) and those who use other more traditional media [56]. Those using bulletin boards were significantly less likely to trust government advice, and were keen to propogate the taking of particular precautions, despite government reassurances. We might anticipate that these anonymous bulletin boards act to further reinforce the messages of “risk junkies”, motivating, for example, many mothers in Eastern Japan to leave their hometowns, or to spend considerable time searching for ‘safe foods’ derived from areas unaffected by radiation. Governmental interventions should aim to combat such concerns where appropriate, fully engaging in the use of such non-traditional media.

### Limitation and Future Directions

Our study had several limitations. Students may not represent the wider Japanese community, who may be differentially impacted by the earthquake. Young mothers, for example, may be a particular risk group following the Fukushima nuclear incident, with large numbers of such mothers leaving areas such as Tokyo and Chiba [57]. Our results are cross-sectional, precluding the analysis of reciprocal pathways; traumatic life events can challenge an individual’s “assumptive world”, and in themselves modify individual values [58]. As indicated above, culture is likely to help frame risk perceptions [59]; future work could be profitably conducted in other cultural settings where earthquakes (if not nuclear incidents) are common.

Further outcomes can also profitably be explored, particularly given the continuing challenges faced by the huge number of refugees created by the March 11^th^ earthquake and tsunami (estimated to number around a third of a million persons) [60]. New research is needed into how such individuals actively utilize social networks to help cope with their stresses in an ambiguous situation, where risk may be amplified through certain social interactions [17]. Risk perceptions are liable to have important socio-economic consequences: new adaptations to a perceived reality (e.g. widespread contamination) can have important financial implications on food supply chains, even when the objective risks are low [38]. Building expanded models of risk perception and its outcomes are likely to be of increasing value as Japan experiences continuing challenges following the March 2011 earthquake.

## Supporting Information

Figure S1 **Original conceptual model.** Note: For the poor-fit original model, we tested all the regression paths from predictors to mediators, then from mediators to outcomes variables. However this fully mediated model does not fit well (see text above). For parsimony purpose, we did not draw all the regression paths but instead use two large arrows to illustrate the structural relationships.
(TIF)

Figure S2 **Baseline model for all respondents.** Note: *p<.05, **p<.01, ***p<.001. D0– D4 denote disturbance terms. D0 and D1 are correlated, whereas D2, D3 and D4 are correlated with each other. E. Norm = earthquake normative concern, Conserv = conservation scores, N. Norm = nuclear normative concern, N. Trust = trust in governmental nuclear advice, N. Contr. = control over safety, E. Risk and N. Risk = risk of an earthquake (or nuclear event) affecting safety. Nuclear acts are “avoid certain foods and drinks,” “avoid going outside,” and “wear masks.” Quake acts’ are “kept an emergency kit’ and “modifying houses.” Leave is “considered leaving Japan.”
(TIF)

Table S1 **Residual matrices for the three study sites.**
(DOCX)
