# Supplementary figures and images for: Modelling Psychological Responses to the Great East Japan Earthquake and Nuclear Incident
Source: PLoS One. 2012 May 30;7(5):e37690. doi: 10.1371/journal.pone.0037690 (PMC3364293; doi:10.1371/journal.pone.0037690)

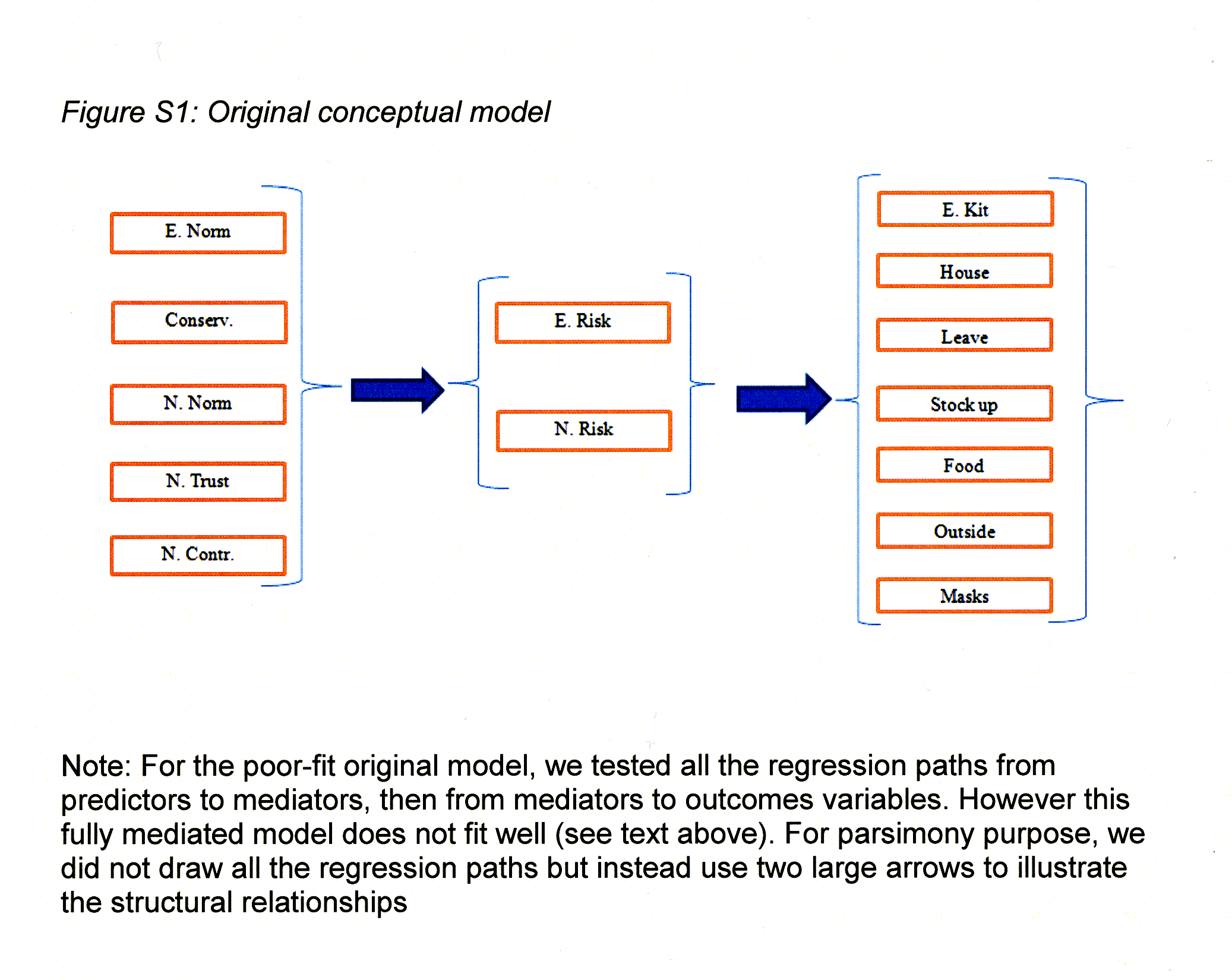

Supplement: Figure S1 — Original conceptual model. Note: For the poor-fit original model, we tested all the regression paths from predictors to mediators, then from mediators to outcomes variables. However this fully mediated model does not fit well (see text above). For parsimony purpose, we did not draw all the regression paths but instead use two large arrows to illustrate the structural relationships. (TIF) [file pone.0037690.s001.tif]

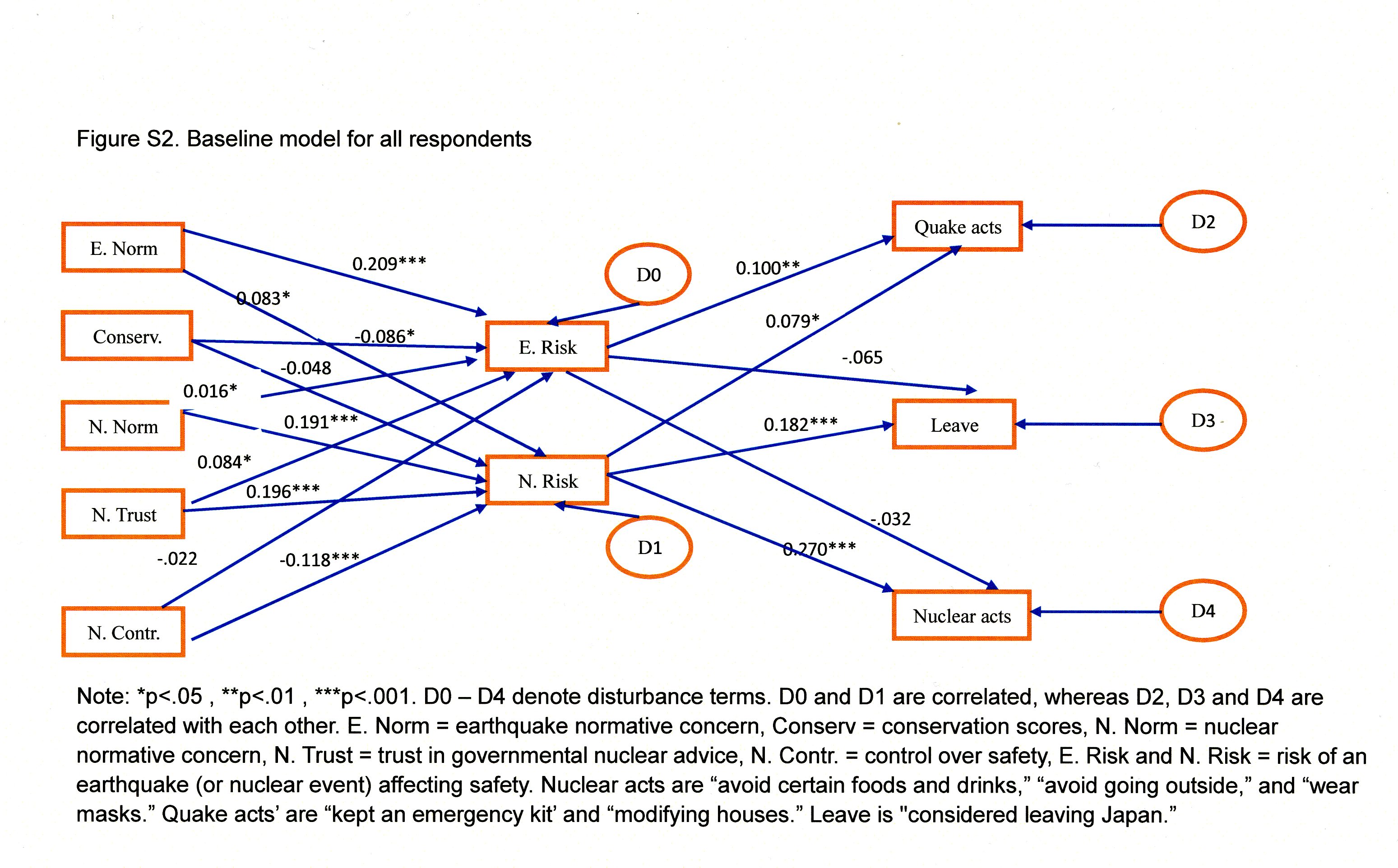

Supplement: Figure S2 — Baseline model for all respondents. Note: *p<.05, **p<.01, ***p<.001. D0– D4 denote disturbance terms. D0 and D1 are correlated, whereas D2, D3 and D4 are correlated with each other. E. Norm = earthquake normative concern, Conserv = conservation scores, N. Norm = nuclear normative concern, N. Trust = trust in governmental nuclear advice, N. Contr. = control over safety, E. Risk and N. Risk = risk of an earthquake (or nuclear event) affecting safety. Nuclear acts are “avoid certain foods and drinks,” “avoid going outside,” and “wear masks.” Quake acts’ are “kept an emergency kit’ and “modifying houses.” Leave is “considered leaving Japan.” (TIF) [file pone.0037690.s002.tif]
