# Supplementary material for: Modelling Psychological Responses to the Great East Japan Earthquake and Nuclear Incident
Source: PLoS One. 2012 May 30;7(5):e37690. doi: 10.1371/journal.pone.0037690 (PMC3364293; doi:10.1371/journal.pone.0037690)
Supplement: Table S1 — Residual matrices for the three study sites. (DOCX) [file pone.0037690.s003.docx]

Table S1: Residual Matrices.

Residual Matrix: Miyagi

|  | control | trust | N_norm | Conserv. | E_norm | E. risk | N. risk | N. act | leave | E. act |
| --- | --- | --- | --- | --- | --- | --- | --- | --- | --- | --- |
| control | 0 |  |  |  |  |  |  |  |  |  |
| trust | -0.065 | 0 |  |  |  |  |  |  |  |  |
| N_norm | 0.018 | 0.006 | 0 |  |  |  |  |  |  |  |
| Conserv. | -0.037 | -0.035 | -0.148 | 0 |  |  |  |  |  |  |
| E. norm | -0.01 | 0.012 | 0.001 | -0.129 | 0 |  |  |  |  |  |
| E. risk | -0.022 | 0.057 | 0.001 | -0.021 | 0.005 | 0 |  |  |  |  |
| N. risk | -0.016 | 0.037 | 0 | -0.039 | 0.01 | 0.016 | 0 |  |  |  |
| N. act | 0.012 | 0.023 | 0.008 | -0.075 | 0.019 | -0.014 | 0.001 | 0 |  |  |
| leave | 0.032 | -0.012 | 0.012 | 0.063 | 0.022 | 0.033 | 0.002 | 0.008 | 0 |  |
| E. act | -0.009 | 0.013 | 0.002 | -0.005 | 0.002 | 0 | 0.012 | 0 | 0.005 | 0 |

Residual Matrix: Tokyo

|  | control | trust | N_norm | Conserv. | E_norm | E. risk | N. risk | N. act | leave | E. act |
| --- | --- | --- | --- | --- | --- | --- | --- | --- | --- | --- |
| control | 0 |  |  |  |  |  |  |  |  |  |
| trust | -0.001 | 0 |  |  |  |  |  |  |  |  |
| N_norm | -0.094 | 0.009 | 0 |  |  |  |  |  |  |  |
| Conserv. | -0.057 | -0.05 | -0.109 | 0 |  |  |  |  |  |  |
| E. norm | -0.049 | 0.019 | 0.001 | -0.18 | 0 |  |  |  |  |  |
| E. risk | -0.026 | 0.064 | 0.001 | -0.026 | 0.03 | 0 |  |  |  |  |
| N. risk | -0.024 | 0.037 | 0.022 | -0.023 | 0.064 | 0.027 | 0 |  |  |  |
| N. act | -0.014 | 0.022 | 0.024 | -0.03 | 0.05 | -0.002 | 0.008 | 0 |  |  |
| leave | 0.019 | 0.016 | 0.047 | 0.042 | 0.013 | -0.032 | 0.003 | 0.012 | 0 |  |
| E. act | -0.014 | -0.006 | 0.035 | -0.073 | 0.005 | 0.001 | 0.007 | 0.008 | 0 | 0 |

Residual Matrix: Western Japan

|  | control | trust | N_norm | Conserv. | E_norm | E. risk | N. risk | N. act | leave | E. act |
| --- | --- | --- | --- | --- | --- | --- | --- | --- | --- | --- |
| control | 0 |  |  |  |  |  |  |  |  |  |
| trust | -0.02 | 0 |  |  |  |  |  |  |  |  |
| N_norm | -0.019 | 0.005 | 0 |  |  |  |  |  |  |  |
| Conserv. | -0.006 | 0.013 | -0.12 | 0 |  |  |  |  |  |  |
| E. norm | 0.003 | 0.009 | 0.001 | -0.115 | 0 |  |  |  |  |  |
| E. risk | 0.011 | 0.029 | 0.051 | -0.015 | 0.015 | 0 |  |  |  |  |
| N. risk | 0.002 | 0.018 | 0.027 | -0.012 | 0.021 | 0.009 | 0 |  |  |  |
| N. act | 0.003 | 0.018 | 0.004 | -0.043 | 0.021 | -0.009 | 0 | 0 |  |  |
| leave | 0.001 | 0.019 | 0.018 | -0.005 | 0.01 | -0.022 | 0 | 0.001 | 0 |  |
| E. act | 0.01 | 0.013 | -0.002 | -0.001 | 0.002 | 0 | -0.005 | 0 | -0.001 | 0 |
